# Causes of Decay of Teeth

**Published:** 1876-08

**Authors:** S. P. Cutler

**Affiliations:** Memphis, Tenn.


### CAUSES OF DECAY OF TEETH.

BY S. P. CUTLER, M. D., D. D. S., MEMPHIS, TENN.

Why teeth decay now more than years gone by, is a subject that has elicited a great deal of attention of late. On noticing an article in the Boston *Chemical Journal*, November, 1875, entitled, "The Physiological Effects of the Water we Drink," it occurred to me that some good hints might be taken as to the etiology of dental caries. The article says: "Some interesting experiments have been made in France by M. Papillon, with a view to test the action of certain phosphates, administered in distilled water, on the boney structures of various animals. These were virtually experiments on artificial drinking water, and they prove that mineral water in dilute solution is capable of being assimilated by the body, the structure of which may thus be materially modified. They think the health of communities may be greatly benefited in physical development by altering the chemical composition of the water supply. The very composition of our bones is affected by the quality of the water we drink. If the waters contain lime, that substance will be taken up and will appear as phosphate of lime in the bones; if it contains strontia or magnesia, these will appear as phosphates of these; if the water is deficient in mineral, there will be a corresponding deficiency in the constitution of the bones. It is said that such instances have occurred in Holland in districts where the inhabitants can obtain only rain water for drinking purposes. This fact probably combined with an absence of lime in their food occasions a softening and distortion of the bones of the body.

This report thinks that by altering a water supply it might be possible to alter the physical organization of a population, and thinks the examination of the bones of a by-gone generation might show the character of the water they drank." Experiments on cattle were made, giving the effects of contamination of organic matter and sulphates, showing conclusively the effects of contamination. On reading the above article it forcibly impressed the idea that the almost unanimous use of cistern water, in cities and large towns in this southern country, for drinking and cooking purposes, might be noted as one of the many causes of bad teeth among children, as they drink a good deal of water; in hot weather ice is used freely.

The hydrant water supply in the more northern cities may be more favorable. In New York, Croton water; in Philadelphia, Schuylkill; in Boston, Cochituate; in Baltimore river water; in Pittsburg, river water; in Buffalo, lake water; in Cincinnati and Louisville, Ohio river water; in Chicago, lake water; in St. Louis, river water; in Nashville, Cumberland river and cistern; in Memphis, only cistern, also New Orleans and other southern cities.

In the country districts in this country, spring and well water is chiefly used, and country children have better teeth, as a rule, and water may be one factor in causation.

In the country, children use more sweet milk, and less sugar and superfine flour than in the cities; take more out door and muscular exercise; go to bed earlier and get up earlier than in the cities, and have better health, bones, muscles and teeth. What is called hard water may be rather unhealthy to drink, by causing bowel affections, but certainly the excess of lime must be favorable for teeth development.

I know full well that when I was a boy, cistern water was never heard of, and I seldom heard any complaints among my associates about diseased teeth, and no dentist was needed to extract our deciduous teeth, we did that with a string, rather as an amusement than otherwise. We knew nothing about laughing gas to kill pain for such purposes, nothing was ever heard about children being nervous. Our public schools develop only intellect; they break the proper physical balance between mental and physical. But where is the remedy? The teeth of negroes show a marked difference in town and country, good in the country bad in the large towns; their living and habits are different. The other day operating for a German lady who was raised in Germany, who has only two teeth decayed, remarked that she believed drinking rain water in Memphis caused her children to have bad teeth; said the water did not contain what the teeth wanted; her daughter, fifteen, has lost the lower six year molars, and the upper ones badly decayed; said they did not drink any rain water where she was raised, nor use ice. She further stated that they did not eat so much hot food in her country; they use a great deal of sweet milk and brown bread. I have often heard similar statements from other foreigners, we might learn a lesson from them.

